# The long non-coding RNA *SNHG12* promotes gastric cancer by activating the phosphatidylinositol 3-kinase/AKT pathway

**DOI:** 10.18632/aging.102493

**Published:** 2019-12-05

**Authors:** Rui Zhang, Yuan Liu, Hui Liu, Wei Chen, Hui-Ning Fan, Jing Zhang, Jin-Shui Zhu

**Affiliations:** 1 Department of Gastroenterology, Shanghai Jiao Tong University Affiliated Sixth People’s Hospital, Shanghai 200233, China

**Keywords:** lncRNA *SNHG12*, gastric cancer, tumorigenesis, PI3K, AKT

## Abstract

Long non-coding RNAs contribute to the development of human cancers. We compared the long non-coding RNA levels in gastric cancer (GC) and para-cancerous tissues in the Gene Expression Omnibus, and found that small nucleolar RNA host gene 12 (*SNHG12*) was upregulated in GC tissues. Fluorescence in situ hybridization confirmed that *SNHG12* is overexpressed in GC tissues. We then used data from The Cancer Genome Atlas to assess the association of *SNHG12* expression with the clinicopathological characteristics and prognosis of GC patients and found that higher *SNHG12* expression was associated with a greater tumor invasion depth and poorer survival. *In vitro*, silencing *SNHG12* suppressed GC cell proliferation, migration and invasion, but induced apoptosis and cell cycle arrest. Overexpressing *SNHG12* had the opposite effects. In xenografted mice, knocking down *SNHG12* reduced GC tumor growth. Taken together, cancer pathway microarray and bioinformatics analyses, RNA pulldown assays, Western blotting and immunohistochemistry revealed that *SNHG12* induces GC tumorigenesis by activating the phosphatidylinositol 3-kinase/AKT pathway. *SNHG12* may thus be a useful marker for predicting poor survival in GC patients.

## INTRODUCTION

According to the global cancer statistics released in 2018, gastric cancer (GC) is the fifth most commonly diagnosed cancer type and the third leading cause of cancer-related mortality worldwide [1]. Although the popularization of gastroscopy has improved the survival of GC patients, the prognosis of patients with advanced GC remains poor due to tumor metastasis and recurrence [2]. The identification of markers associated with tumorigenesis and cancer progression may enable the early detection of GC.

Whole-genome sequencing has revealed that over 90% of the genome is actively transcribed, but 98% of the transcripts have no potential to encode proteins [3]. Among these non-coding transcripts, long non-coding RNAs (lncRNAs), which include at least 200 nucleotides, have attracted more and more attention [4]. LncRNAs have been found to regulate gene splicing, chromatin organization and gene transcription [5]. The dysregulation of lncRNAs is involved in a range of diseases, including cancers [6–8]. LncRNAs have been reported to promote the growth, metastasis and drug resistance of cancer cells [9–11].

Aberrant lncRNA expression is associated with GC development, and lncRNAs may serve as tumor suppressors or oncogenes in GC [12]. For instance, the lncRNAs *HOTTIP*, *GAPLINC* and *AK023391* are independent predictors of poor survival and are also potential diagnostic markers in GC patients [13–15]. The lncRNAs *PVT1*, *GClnc1* and *AGAP2-AS1* have been reported to promote the proliferation and invasion of GC [16–18], while *linc00261* and *MEG3* serve as tumor suppressors in GC [19, 20]. LncRNAs stimulate the pathogenesis of GC through their participation in key signaling pathways. For example, the lncRNAs *ADAMTS9-AS2*, *HOTAIR* and *ZEB2-AS1* induce GC tumorigenesis through the phosphatidylinositol 3-kinase (PI3K)/AKT, NF-κB and Wnt/β-catenin signaling pathways, respectively [21–23].

Herein, we found that increased expression of the lncRNA *SNHG12* reduced the overall survival of GC patients and promoted GC tumorigenesis by activating the PI3K/AKT pathway. Thus, *SNHG12* could be used as a biomarker to predict poor survival in GC patients.

## RESULTS

### *SNHG12* is upregulated in human GC and is associated with a poor prognosis

**Figure 1:**
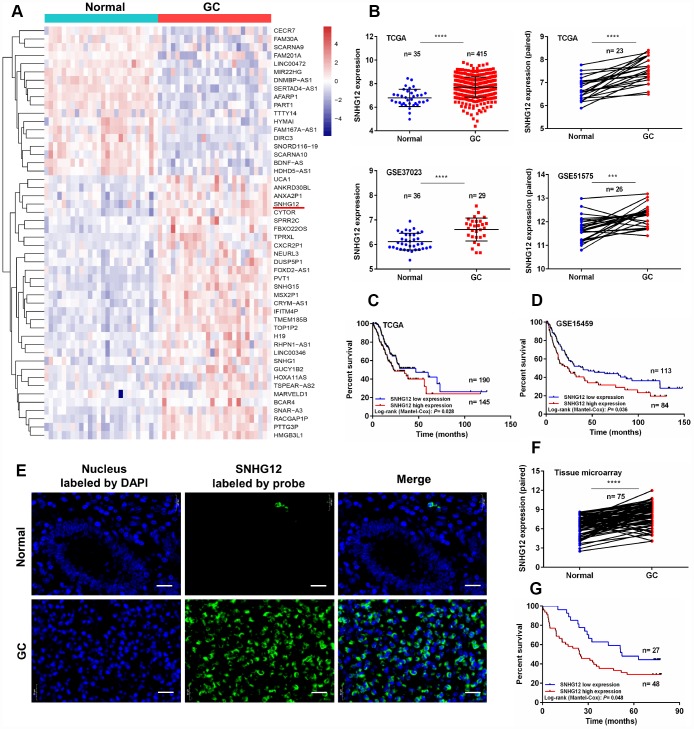
***SNHG12* is upregulated in human GC and is associated with a poor prognosis.** (**A**) Hierarchical heat map of differentially expressed genes between GC and para-cancerous tissues from the Gene Expression Omnibus database. Blue denotes downregulated genes and red denotes upregulated genes. (**B**) *SNHG12* levels were detected in unpaired and paired GC tissues from TCGA, GSE37023 and GSE51575 cohorts. (**C**, **D**) Kaplan-Meier analysis of the association between *SNHG12* expression and overall survival in GC patients, based on TCGA and GSE15459 cohorts. (**E**, **F**) FISH analysis of the subcellular localization and expression of *SNHG12* in GC and para-cancerous tissues. The nucleus was labeled with DAPI (blue) and *SNHG12* was labeled with a probe (green). Scale bar: 20 μm. (**G**) Kaplan-Meier analysis of the association between *SNHG12* expression and overall survival in GC patients, based on the tissue microarray. ***P<0.001, ****P<0.0001.

In this study, we first compared the RNA expression data of GC and adjacent normal tissues from the Gene Expression Omnibus (GSE51575). As expected, various lncRNAs were aberrantly expressed in GC tissues (Figure 1A). We focused our attention on upregulated lncRNAs, as they may be more suitable than downregulated lncRNAs for use as early diagnostic markers in cancer patients [2]. Among the overexpressed lncRNAs in GC tissues, *SNHG12* was highly abundant, and thus was selected for further study. We then analyzed *SNHG12* expression in two independent cohorts downloaded from The Cancer Genome Atlas (TCGA) and GSE37023. *SNHG12* was dramatically upregulated in unpaired and paired cancer tissues compared with para-cancerous tissues, both in GC (Figure 1B) and in other malignancies (Supplementary Figure 1).

Next, we analyzed the correlation between *SNHG12* expression and clinicopathological factors in patients with GC. We used Cutoff Finder to divide the GC patients from TCGA into low and high *SNHG12* expression groups [24]. *SNHG12* expression correlated with the tumor invasion depth (*P* = 0.009), but had no relationship with other clinical factors in GC patients (Table 1). Kaplan-Meier analysis revealed that survival was poorer in GC patients with high *SNHG12* expression (median survival time: 22.50 months) than in those with low *SNHG12* expression (median survival time: 46.90 months) (*P* = 0.028, Figure 1C). Similar results were obtained from a Kaplan-Meier Plotter analysis (*P* = 0.036, Figure 1D) [25].

**Table 1:** The correlation between *SNHG12* expression and clinicopathological factors in GC patients.

**Characteristics**	**Number of cases**	**Expression of *SNHG12***		*P* value*
		**Low**	**High**	
Gender				0.777
male	212	119	93	
female	123	71	52	
Age				0.312
≤ 60	114	69	45	
> 60	221	121	100	
Histological grade				0.234
Low	213	126	87	
Middle + high	122	64	58	
Tumor invasion depth				0.009**
T1	16	4	12	
T2 + T3 + T4	319	186	133	
Lymph node metastasis				0.339
N0	104	63	41	
N1 + N2 + N3	231	127	104	
Distant metastasis				0.873
M0	315	179	136	
M1	20	11	9	
TNM stage				0.457
I + II	151	89	62	
III + IV	184	101	83	

*chi-square test; ** *P* < 0.01, statistically significant results (in bold)

To further explore the prognostic value of *SNHG12* in GC, we conducted a univariate Cox regression analysis of potential survival-associated factors. We found that higher *SNHG12* expression (*P* = 0.029), older age (*P* = 0.008), a greater tumor invasion depth (*P* = 0.030), lymph node metastasis (*P* = 0.001), distant metastasis (*P* = 0.007) and a higher TNM stage (*P* < 0.001) were associated with poorer survival in GC patients. Multivariate Cox regression analysis of these factors revealed that high *SNHG12* expression was a risk factor for poor survival in GC patients (hazard ratio [HR]=1.528, 95% confidence interval [CI]=1.085-2.152, *P* = 0.015, Table 2).

**Table 2:** Univariate and multivariate analyses of clinicopathological factors influencing overall survival in GC patients.

**Risk factors**	**Univariate analysis**			**Multivariate analysis**		
	**HR**	***P* value**	**95% CI**	**HR**	***P* value**	**95% CI**
*SNHG12* expression (low, high)	1.462	0.029*	1.040 - 2.057	1.528	0.015*	1.085 - 2.152
Age (≤ 60, > 60)	1.694	0.008**	1.150 - 2.496	1.871	0.002**	1.263 - 2.769
Tumor invasion depth (T1, T2 + T3 + T4)	8.843	0.030*	1.236 - 63.293			
Lymph node metastasis (N0, N1 + N2 + N3)	2.056	0.001**	1.330 - 3.177			
Distant metastasis (M0, M1)	2.357	0.007**	1.269 - 4.376	1.980	0.036*	1.046 - 3.748
TNM stage (I + II, III + IV)	2.082	< 0.001**	1.437 - 3.017	1.767	0.004**	1.205 - 2.591
Histological grade (low, middle + high)	0.719	0.075	0.501 - 1.033			
Gender (female, male)	1.307	0.157	0.902 - 1.895			

HR: hazard ratio, CI: confidence interval; *P < 0.05, **P < 0.01, statistically significant results (in bold)

To validate the above results, we performed fluorescence in situ hybridization (FISH) analysis to evaluate *SNHG12* expression in tissues from another 75 GC patients. *SNHG12* was predominantly detected in the cytoplasm, and was markedly upregulated in GC tissues (Figure 1E and 1F). *SNHG12* expression correlated with the tumor invasion depth (*P* = 0.021) and TNM stage (*P* = 0.013, Supplementary Table 1), and patients with high *SNHG12* expression exhibited poorer overall survival than those with low *SNHG12* expression (Figure 1G). Univariate and multivariate Cox regression analyses revealed that high *SNHG12* expression predicted poor survival in GC patients (Supplementary Table 2).

### *SNHG12* promotes GC cell growth

**Figure 2:**
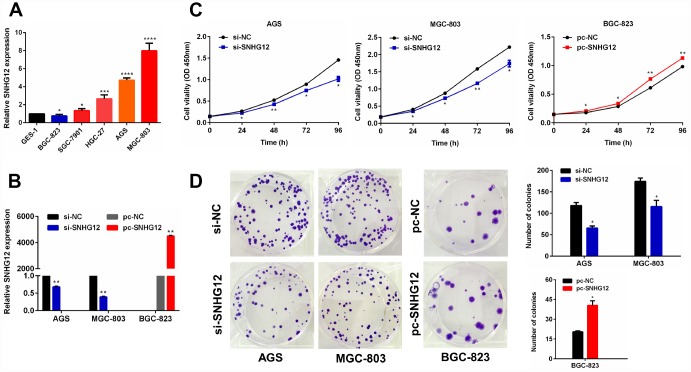
***SNHG12* promotes the growth of GC cells.** (**A**) *SNHG12* levels in GES-1 and GC cell lines were detected by qRT-PCR. (**B**) The transfection efficiencies of si-*SNHG12* in AGS and MGC-803 cells and pc-*SNHG12* in BGC-823 cells were detected by qRT-PCR. (**C**) A CCK-8 assay was used to detect the proliferation of AGS and MGC-803 cells transfected with si-*SNHG12*, and of BGC-823 cells transfected with pc-*SNHG12*. (**D**) A colony formation assay was used to detect the colony formation abilities of AGS and MGC-803 cells transfected with si-*SNHG12*, and of BGC-823 cells transfected with pc-*SNHG12*. Data are shown as the mean ± standard error of the mean (SEM) from at least three experiments. *P**<**0.05, **P**<**0.01, ***P**<**0.001, ****P**<**0.0001.

To determine the function of *SNHG12* in GC cells, we first examined its expression in GC cell lines by performing quantitative real-time PCR (qRT-PCR) analysis. *SNHG12* expression was higher in the AGS and MGC-803 cell lines but lower in the BGC-823 cell line than in the GES-1 (normal) cell line (Figure 2A). Thus, we performed knockdown experiments in AGS and MGC-803 cells, and conducted overexpression experiments in BGC-823 cells. A qRT-PCR analysis confirmed that transfection with small interfering RNA (siRNA) for *SNHG12* (si-*SNHG12*) efficiently silenced *SNHG12* in AGS and MGC-803 cells, while transfection with an overexpression plasmid (pc-*SNHG12*) upregulated *SNHG12* in BGC-823 cells (Figure 2B).

We then performed Cell Counting Kit 8 (CCK-8) and colony formation assays in the transfected cells. The knockdown of *SNHG12* suppressed cell proliferation and colony formation in the AGS and MGC-803 cell lines, while the overexpression of *SNHG12* had the opposite effects in the BGC-823 cell line (Figure 2C and 2D). However, at 18 hours, there was no significant difference in the proliferation of GC cells, regardless of whether *SNHG12* was knocked down or overexpressed (Supplementary Figure 2).

### *SNHG12* promotes the migration and invasion of GC cells

**Figure 3:**
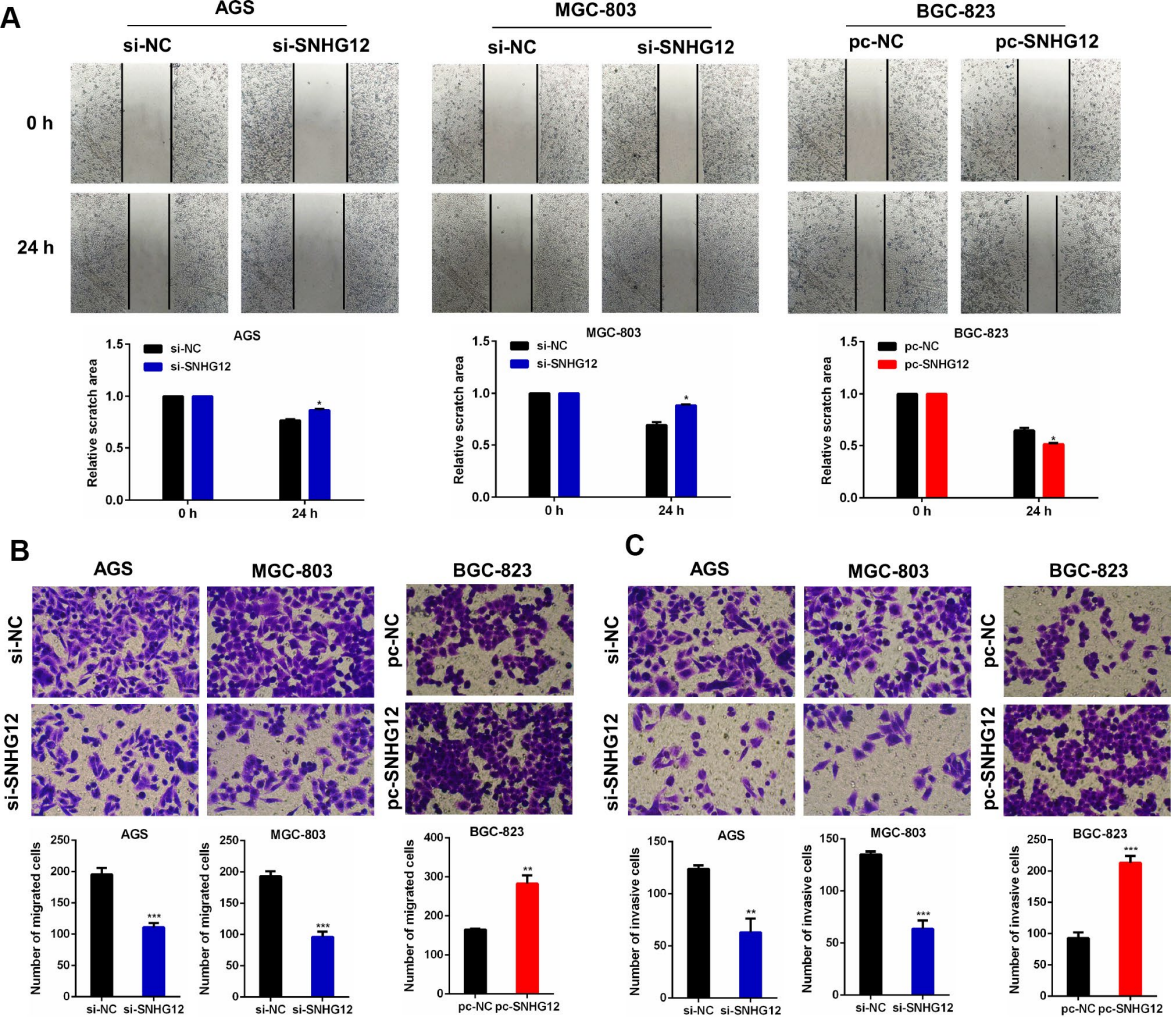
***SNHG12* promotes the migration and invasion of GC cells.** (**A**, **B**) Wound healing and Transwell migration assays were performed to evaluate the migration abilities of AGS and MGC-803 cells transfected with si-*SNHG12,* and of BGC-823 cells transfected with pc-*SNHG12*. (**C**) A Transwell invasion assay was conducted to estimate the invasion abilities of AGS and MGC-803 cells transfected with si-*SNHG12,* and of BGC-823 cells transfected with pc-*SNHG12*. The migratory and invasive cells were stained with Hoechst. Data are shown as the mean ± SEM of at least three experiments. *P<0.05, **P<0.01, ***P<0.001.

To explore the effects of *SNHG12* on GC cell migration and invasion, we performed wound healing and Transwell migration/invasion experiments. The wound healing assay demonstrated that the knockdown of *SNHG12* suppressed the migration of AGS and MGC-803 cells, while the overexpression of *SNHG12* promoted the migration of BGC-823 cells (Figure 3A). Similar results were observed in the Transwell migration assay: the number of cells passing through the polycarbonate membrane decreased after the knockdown of *SNHG12* in AGS and MGC-803 cells, but increased after the overexpression of *SNHG12* in BGC-823 cells (Figure 3B). A Transwell invasion assay revealed that knocking down *SNHG12* substantially lowered the invasive abilities of AGS and MGC-803 cells, while overexpressing *SNHG12* enhanced the invasion of BGC-823 cells (Figure 3C).

### *SNHG12* promotes cell cycle progression and reduces apoptosis in GC cells

**Figure 4:**
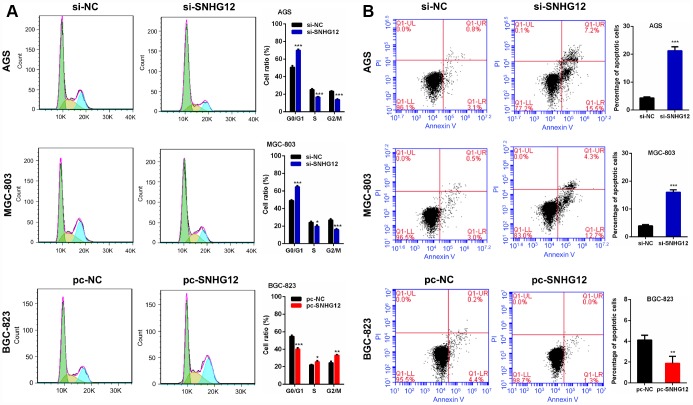
***SNHG12* promotes cell cycle progression and reduces apoptosis in GC cells.** (**A**) The cell cycle distribution of AGS and MGC-803 cells transfected with si-*SNHG12* and BGC-823 cells transfected with pc-*SNHG12*. Green, yellow and blue represent the cells in G0/G1 phase, S phase and G2/M phase, respectively. (**B**) The apoptosis of AGS and MGC-803 cells transfected with si-*SNHG12* and BGC-823 cells transfected with pc-*SNHG12*. Data are shown as the mean ± SEM of at least three experiments. *P<0.05, **P<0.01, ***P<0.001.

The effects of *SNHG12* on cell cycle progression and apoptosis in GC cells were evaluated by flow cytometry. The knockdown of *SNHG12* in AGS and MGC-803 cells increased the number of cells in G0/G1 phase but reduced the number of cells in S phase and G2/M phase. On the other hand, the overexpression of *SNHG12* in BGC-823 cells reduced the number of cells in G0/G1 phase but increased the number of cells in S phase and G2/M phase (Figure 4A). An apoptosis analysis revealed that knocking down *SNHG12* promoted apoptosis in AGS and MGC-803 cells, while overexpressing *SNHG12* inhibited apoptosis in BGC-823 cells (Figure 4B).

### *SNHG12* activates the PI3K/AKT pathway in GC cells

**Figure 5:**
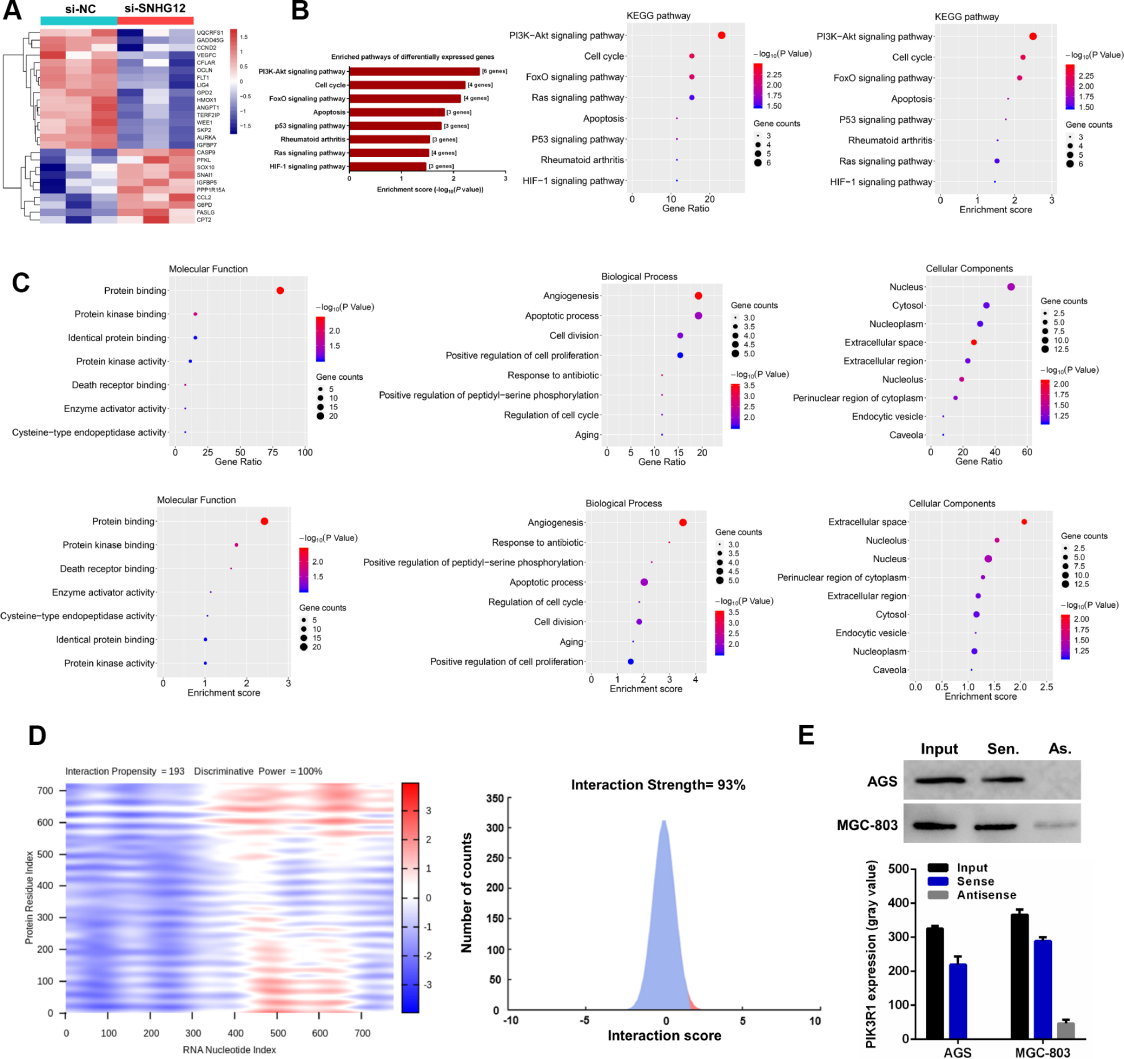
***SNHG12* is involved in the PI3K/AKT pathway in GC cells.** (**A**) Hierarchical heat map of differentially expressed genes between si-NC- and si-*SNHG12*-transfected MGC-803 cells. (**B**) KEGG pathway enrichment analysis revealed that the differentially expressed genes were mainly enriched in eight pathways. (**C**) Gene Ontology analysis of the differentially expressed genes. (**D**) The prediction results demonstrated that *SNHG12* was likely to bind to PIK3R1. (**E**) An RNA pulldown assay was performed to confirm that *SNHG12* could bind to PIK3R1 in AGS and MGC-803 cells.

To comprehensively analyze the pathways regulated by *SNHG12* in GC cells, we conducted a cancer pathway microarray analysis in MGC-803 cells. The microarray contained 90 genes, and 26 of them were differentially expressed (fold-change ≥ 1.5, *P* < 0.05) in the si-*SNHG12* group compared with the negative control siRNA (si-NC) group (Figure 5A). The pathway enrichment of these differentially expressed genes was analyzed online in the Database for Annotation, Visualization and Integrated Discovery (DAVID). Kyoto Encyclopedia of Genes and Genomes (KEGG) pathway analysis on the DAVID website revealed that these 26 genes were mainly enriched in eight signaling pathways. The PI3K/AKT pathway had the highest number of enriched genes and the highest enrichment score (Figure 5B and Supplementary Figure 3).

We also performed a Gene Ontology analysis of the differentially expressed genes, including assessments of the biological process, molecular function and cell component (Figure 5C). The biological process analysis indicated that the differentially expressed genes were mainly involved in angiogenesis, apoptosis, cell proliferation and cell cycle regulation. The principal molecular functions of the genes were protein binding, protein kinase binding and protein kinase activity. The genes were mostly localized to the nucleus and cytosol.

To investigate the mechanism by which *SNHG12* regulates the PI3K/AKT pathway, we first used online tools to predict whether *SNHG12* could directly bind to the regulatory subunit of PI3K (PIK3R1). In such analyses, the interaction strength can range from 0% (non-interacting) to 100% (interacting), and interaction strengths above 50% indicate a propensity for binding. As shown in Figure 5D, the interaction strength between *SNHG12* and PIK3R1 was 93%; thus, there was a high probability that lncRNA *SNHG12* could bind to PIK3R1. An RNA pulldown assay confirmed the ability of *SNHG12* to pull down the PI3K protein (Figure 5E).

**Figure 6:**
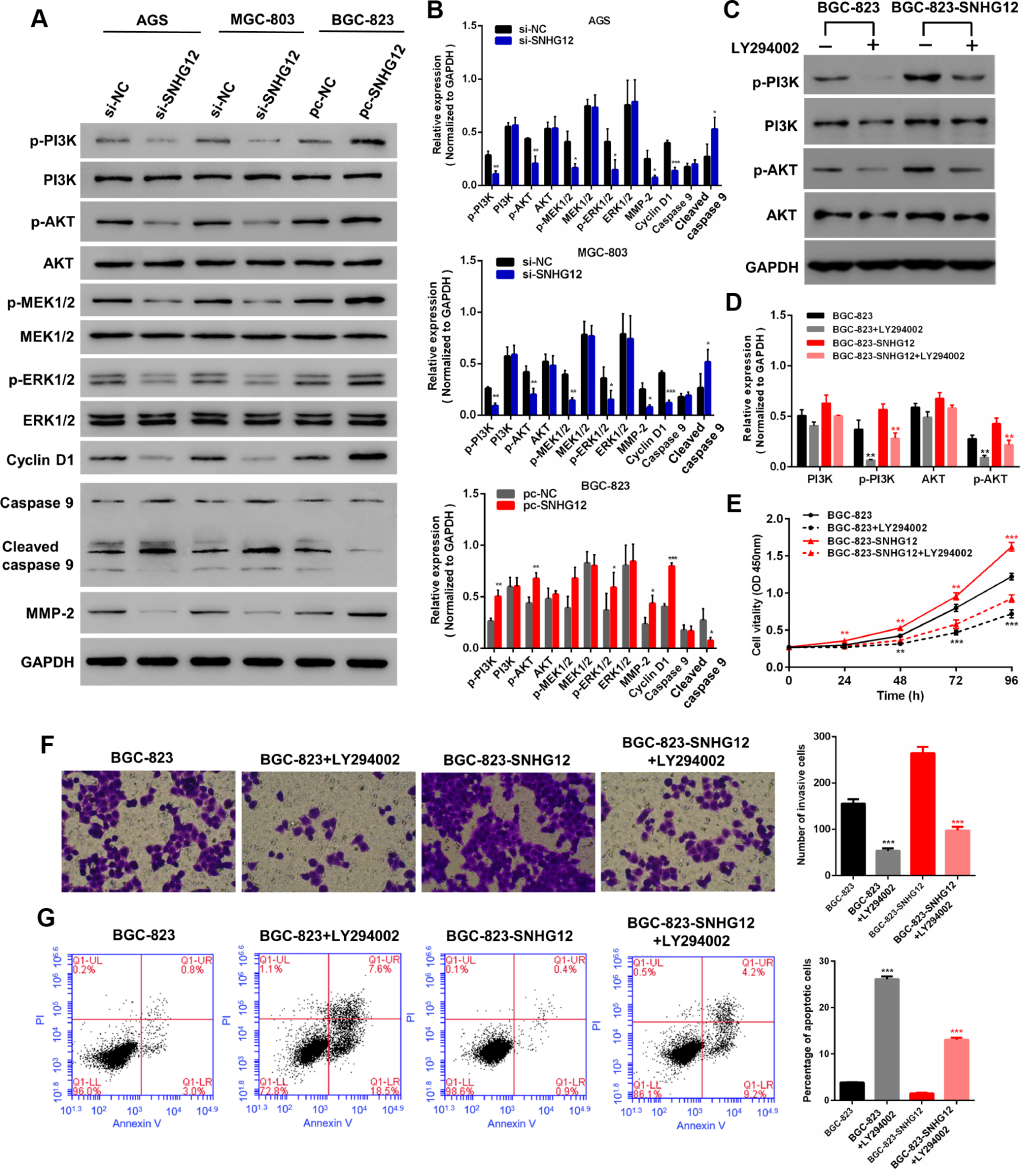
***SNHG12* promotes the progression of GC by activating the PI3K/AKT pathway.** (**A**, **B**) Western blot analysis was performed to detect PI3K/AKT pathway and downstream protein levels in AGS and MGC-803 cells transfected with si-*SNHG12*, and in BGC-823 cells transfected with pc-*SNHG12*. (**C**, **D**) LY294002 inhibited the expression of p-PI3K and p-AKT in BGC-823 and *SNHG12*-overexpressing BGC-823 cells. (**E**) The effects of LY294002 on the proliferation of BGC-823 and *SNHG12*-overexpressing BGC-823 cells were detected with a CCK-8 assay. (**F**) The effects of LY294002 on the invasion of BGC-823 and *SNHG12*-overexpressing BGC-823 cells were detected with a Transwell assay. (**G**) The effects of LY294002 on the apoptosis of BGC-823 and *SNHG12*-overexpressing BGC-823 cells were detected by flow cytometry. Data are shown as the mean ± SEM of at least six experiments. *P<0.05, **P<0.01, ***P<0.001.

We also performed Western blotting to measure the expression of PI3K/AKT pathway proteins known to be associated with cell growth, invasion and apoptosis (Figure 6A and 6B). The knockdown of *SNHG12* in AGS and MGC-803 cells reduced the expression of phosphorylated (p)-PI3K, p-AKT, p-MEK1/2 (mitogen-activated protein kinase kinase 1/2), p-ERK1/2 (extracellular signal-regulated kinase 1/2), cyclin D1 and matrix metalloproteinase (MMP)-2, but increased the expression of cleaved caspase 9, while the overexpression of *SNHG12* in BGC-823 cells had the opposite effects. However, the total levels of PI3K, AKT, MEK1/2 and ERK1/2 were not affected by *SNHG12* expression in GC cells.

To further explore the involvement of the PI3K/AKT pathway in the tumorigenesis of GC, we used LY294002 to inhibit the activity of PI3K in wild-type and *SNHG12*-overexpressing BGC-823 cells. LY294002 inhibited the expression of both p-PI3K and p-AKT (Figure 6C and 6D). A CCK-8 assay demonstrated that inhibiting the PI3K/AKT pathway suppressed GC cell proliferation (Figure 6E). Consistently, GC cell invasion decreased and apoptosis increased when the PI3K/AKT pathway was inhibited (Figure 6F and 6G).

### Knockdown of *SNHG12* inhibits xenograft tumor growth

**Figure 7:**
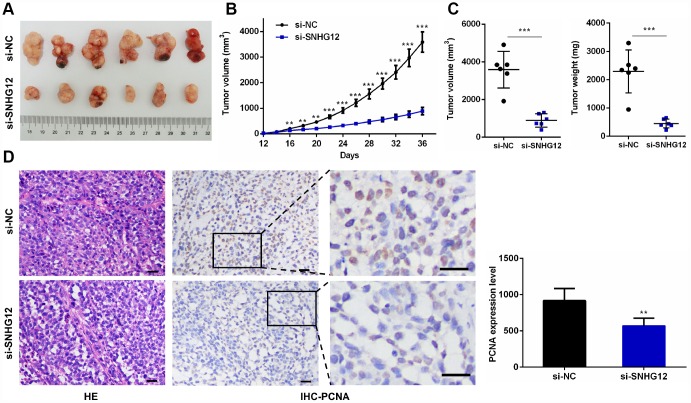
**Knocking down *SNHG12* inhibits xenograft tumor growth.** (**A**) Representative images of xenograft tumors from the si-NC and si-*SNHG12* groups. (**B**) The tumor growth curves of the si-NC and si-*SNHG12* groups. (**C**) The tumor volumes and tumor weights of the si-NC and si-*SNHG12* groups. (**D**) Representative images of hematoxylin and eosin staining and PCNA immunostaining of xenograft tumor samples from the si-NC and si-*SNHG12* groups (scale bar = 200 μm), and semi-quantitative results of PCNA immunostaining. Data are shown as the mean ± SEM of at least six experiments. **P<0.01, ***P<0.001.

Having identified the growth-promoting effects of *SNHG12* in GC cells *in vitro*, we sought to verify these results *in vivo*. AGS cells transfected with si-NC or si-*SNHG12* were used to establish a subcutaneous xenograft tumor model in nude mice. The tumor volumes and weights were markedly lower in the si-*SNHG12* group than in the si-NC group (Figure 7A–7C). Hematoxylin and eosin staining revealed that the number of tumor cells was lower in the si-*SNHG12* group than in the si-NC group, and immunohistochemistry analysis revealed that the protein expression of proliferating cell nuclear antigen (PCNA) was significantly lower in the si-*SNHG12* group than in the si-NC group (Figure 7D).

## DISCUSSION

Small nucleolar RNA host genes (SNHGs), a subgroup of lncRNAs, are associated with the progression of various malignancies, including GC. For instance, *SNHG5* is a tumor suppressor in GC [26, 27], *SNHG8* predicts a poor prognosis in GC patients [28] and *SNHG17* induces the development of GC [29]. Previous studies have indicated that *SNHG12* promotes the tumorigenesis of multiple cancers, including hepatocellular carcinoma, glioma and breast cancer [30–32]. However, little has been known about the clinical value of *SNHG12* in GC patients. Through data mining and tissue microarray analysis, we discovered that *SNHG12* was differentially expressed in GC and adjacent normal tissues, and by evaluating additional GC tissues, we confirmed that *SNHG12* was markedly upregulated in GC. We also found that higher expression of *SNHG12* was associated with a greater tumor invasion depth and poorer survival in GC patients. Thus, in accordance with its tumorigenic role in other cancers [30–32], *SNHG12* may be a promising biomarker for gastric cancer.

Subsequently, we performed a range of functional experiments *in vitro* and *in vivo* to investigate the effects of *SNHG12* on GC cells. Our findings demonstrated that silencing *SNHG12* suppressed GC cell proliferation, colony formation, migration, invasion, cell cycle progression and *in vivo* tumorigenesis, and promoted apoptosis and cell cycle arrest, while overexpressing *SNHG12* had the opposite effects. Thus, both clinical data and functional experiments suggested that *SNHG12* is an oncogenic factor in GC.

PI3K belongs to a family of lipid kinases that can phosphorylate the 3’-OH group of the inositol ring in inositol phospholipids [33]. AKT is a member of the AGC kinase family, which also includes adenosine/ guanosine monophosphate-activated protein kinases and protein kinase C [34]. The PI3K/AKT signaling network influences numerous processes that are dysregulated in cancer, including genomic stability, cell survival, metabolism, cell cycle progression and cell motility [35]; thus, this pathway is frequently overactivated in human malignant tumors [36]. Various lncRNAs (including *DANCR*, *SCAT7* and *AK023391*) have been reported to stimulate the PI3K/AKT pathway in cancers [15, 37, 38].

In our FISH analysis, we detected *SNHG12* mainly in the cytoplasm of GC cells, indicating that *SNHG12* may regulate signal transduction in GC. Our cancer pathway microarray and bioinformatic analyses suggested that *SNHG12* regulates the PI3K/AKT pathway. An RNA pulldown assay confirmed that *SNHG12* could bind directly to PI3K. Thereafter, our Western blot analyses revealed that the phosphorylated levels (but not the total levels) of PI3K and AKT were significantly reduced after *SNHG12* knockdown and elevated after *SNHG12* overexpression, suggesting that *SNHG12* activates this pathway by inducing the phosphorylation of PI3K and AKT. Thus, we deduced that *SNHG12* increased the phosphorylation of PI3K by directly binding to it.

**Figure 8:**
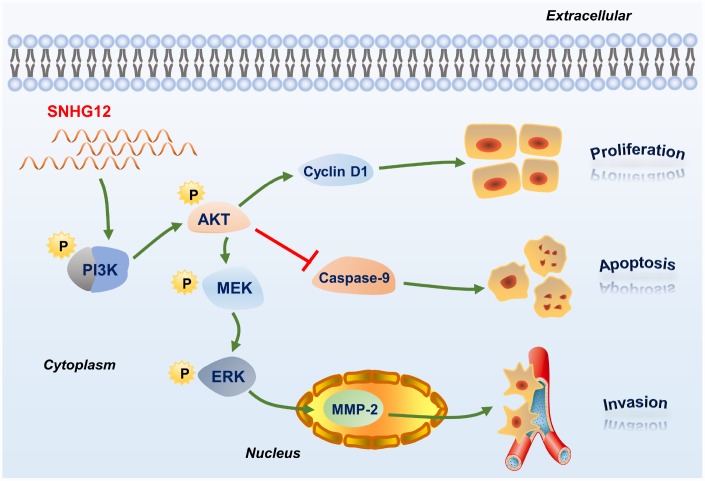
**Schematic diagram of the regulatory mechanism of *SNHG12* in GC cells.**

The levels of PI3K/AKT pathway proteins and downstream proteins were also altered after the knockdown or overexpression of *SNHG12*. These proteins, including p-MEK1/2, p-ERK1/2, cyclin D1, caspase 9 and MMP-2, are widely known to be essential for cell proliferation, apoptosis and invasion. In addition, inhibiting the PI3K/AKT pathway in *SNHG12*-overexpressing BGC-823 cells suppressed cell proliferation and invasion and induced apoptosis. Therefore, we speculated that *SNHG12* promoted GC tumorigenesis by activating the PI3K/AKT pathway (Figure 8). In view of these findings, *SNHG12* overexpression could be used as a biomarker of poor survival in GC patients.

## MATERIALS AND METHODS

### RNA expression profiling

The RNA expression profiling data used to explore differentially expressed genes between GC and adjacent normal tissues were downloaded from the Gene Expression Omnibus. Differentially expressed genes (fold change ≥ 1.5, *P* < 0.05) were identified from these raw data by R 3.5.3 software (Lucent Technologies, NJ, USA).

### Clinical data

The relative *SNHG12* levels and clinical data from 335 GC patients were downloaded from TCGA RNA-seq database. A GC tissue microarray (No. HStmA180Su09), which included 75 GC tissue samples and the corresponding para-cancerous tissues, was purchased from Shanghai Outdo Biotech Co., Ltd (Shanghai, China). This study was approved by the Ethics Committee of Shanghai Sixth People’s Hospital.

### Cell culture

Normal gastric epithelial cells (GES-1) and human GC cell lines (SGC-7901, HGC-27, BGC-823, MGC-803 and AGS) were stored in the Digestive Disease Laboratory of Shanghai Sixth People’s Hospital. The cells were cultured in RPMI-1640 medium (KeyGen, Nanjing, China) with 10% fetal bovine serum (Gibco, MA, USA) and maintained in a humidified atmosphere of 5% CO_2_ at 37 °C.

### FISH

A digoxigenin-labeled DNA probe for *SNHG12* (5’-TCTTGATGGGACCGTTTTATCCGCCTGTCAGCC-3’) was synthesized by Sangon Biotech (Shanghai, China). The tissue microarray was placed in an oven at 60 °C for one hour to bake the wax, and was then dewaxed with xylene. A DNA probe hybridization solution was added to the tissue microarray. After hybridization, the slide was washed in 5×, 1× and 0.2× sodium saline citrate for five minutes each. The tissue microarray was incubated with an anti-digoxigenin horseradish peroxidase conjugate (NEF832001EA, Perkin Elmer) at 4 °C overnight, and was then incubated with a tyramide signal amplification fluorescent reaction solution (NEL701001KT, Perkin Elmer) for 30 minutes. Lastly, the microarray was sealed with tablets containing 4′,6-diamidino-2-phenylindole (DAPI).

The tissue microarray was scanned with a Pannoramic MIDI (3D HISTECH, Budapest, Hungary), and the images were acquired with CaseViewer software (3D HISTECH). Image-Pro Plus 6.0 software (Media Cybernetics, MD, USA) was used to obtain the integrated optical density of the *SNHG12* signal. The results were obtained from at least four different areas with > 70 cells measured per area [39].

### Vector construction and transfection

*SNHG12* siRNAs were synthesized by GenePharma (Shanghai, China). Their sequences were 5’-GCUUGUUUCUGGAGCUGUGTT-3’ (sense) and 5’-CACAGCUCCAGAAACAAGCTT-3’ (antisense). Full-length cDNA from *SNHG12* was synthesized and cloned into the vector pcDNA3.1 (GenePharma). The siRNAs and plasmid vectors were transfected into GC cells by means of Lipofectamine 2000 (Invitrogen, CA, USA) according to the manufacturer’s protocols.

### qRT-PCR

Total RNA was isolated with an RNAeasy™ Animal RNA Isolation Kit (Beyotime, Shanghai, China). Subsequently, the total RNA was reverse-transcribed to cDNA with a PrimeScript™ Reverse Transcription Kit (TakaRa, Japan), and qPCR was conducted with the TB Green™ Premix Ex Taq™ II Kit (TakaRa) according to the manufacturer’s instructions. Primers specific for *SNHG12* (Forward: 5’-TCTGGTGATCGAGGACTTCC -3’, Reverse: 5’-ACCTCCTCAGTATCACACACT-3’) were synthesized by Sangon Biotech. Glyceraldehyde 3-phosphate dehydrogenase (*GAPDH*) was used as the internal control.

### CCK-8 assay

GC cells (5000/well) transfected with *SNHG12* siRNAs or plasmids were incubated in 96-well plates. The CCK-8 reagent (10 μL, Dojindo, Japan) was added to each well at 0, 18, 24, 48, 72 or 96 hours. The cells were incubated for one hour, and a microplate reader was used to measure the optical density at 450 nm.

### Colony formation assay

GC cells (200/well) were placed in six-well plates and cultured for 15 days. Then, 4% paraformaldehyde and 1% crystal violet were used to fix and stain the cells, respectively. The number of colonies containing ≥ 50 cells was counted under a microscope.

### Wound-healing assay

GC cells (1.5×10^6^/well) were placed in six-well plates and cultured for 24 hours. After the cells had adhered to the plate, they were scratched with a sterile 100-μL pipette tip. The scratch area was measured with Image-Pro Plus software.

### Transwell migration and invasion assay

Transwell chambers with 8-μm pores (Corning, NY, USA) were used to assess the migratory abilities of GC cells. For the migration assay, 100 μL of serum-free medium containing 5×10^5^ cells was added to the upper chamber, while 600 μL of complete medium was added to the lower chamber. The invasion assay resembled the migration assay, but the Transwell chambers were coated with Matrigel (Becton Dickinson, NJ, USA). After the chambers had been incubated for 18 h, the migratory or invasive cells were fixed, stained with Hoechst and photographed under a fluorescence microscope.

### Cell cycle analysis

Transfected GC cells were collected by centrifugation and then fixed with cold 70% ethanol at 4 °C overnight. Then, RNase A (1 mg/mL, Solarbio, Beijing, China) was used to remove RNA, and the cells were stained with propidium iodide (50 μg/mL) at 37 °C in the dark for 30 minutes. The fluorescence signal excited at 488 nm was detected with a flow cytometer (Becton Dickinson).

### Apoptosis assay

An apoptosis assay was performed with an Annexin V-FITC Apoptosis Detection Kit (Beyotime) according to the instructions of the manufacturer. The percentage of apoptotic cells was analyzed on a flow cytometer.

### Cancer pathway microarray analysis

To acquire all-sided results on *SNHG12*-associated signaling pathways, we used a human cancer pathway microarray (No. PAHS-033Z, Qiagen, Dusseldorf, Germany) to identify differentially expressed genes between si-*SNHG12-* and si-NC-transfected MGC-803 cells. We then used the online DAVID tool for KEGG and Gene Ontology analyses of the differentially expressed genes.

### Binding prediction and RNA pulldown assay

Before performing the RNA pulldown assay, we first used the online tools catRAPID and PRIDB to predict whether lncRNA *SNHG12* could bind to PI3K protein. The links of the websites and the sequences of *SNHG12* and PIK3R1 can be found in the supplementary materials.

For the RNA pulldown assay, the Biotin RNA Labeling Mix (Roche, Basel, Switzerland) was used to transcribe and label sense- or antisense-*SNHG12* from the pcDNA 3.1-*SNHG12* vector *in vitro*. An RNA structure buffer (Thermo, MA, USA) was used to induce secondary structure formation from the biotin-labeled RNAs. Streptavidin beads (Thermo) were washed three times with 500 μL of RNA immunoprecipitation wash buffer (Thermo) and then added to the biotinylated RNAs at 4 °C overnight. The overnight mixture was separated by a magnetic field so that streptavidin bead-RNA complexes could be obtained. Then, lysates of AGS or MGC-803 cells were added to the complexes and incubated on a rotator at room temperature for one hour. The incubated mixture was again separated with a magnetic field so that streptavidin bead-RNA-protein complexes could be obtained. These complexes were used for Western blotting with a PIK3R1 primary antibody.

### Western blot assay

Total protein was extracted from GC cells with lysis buffer and boiled in loading buffer for five minutes. Subsequently, the total protein was separated by sodium dodecyl sulfate polyacrylamide gel electrophoresis and transferred to polyvinylidene difluoride membranes. Primary antibodies against p-PI3K, PI3K, p-AKT, AKT, p-MEK1/2, MEK1/2, p-ERK1/2, ERK1/2, cyclin D1, caspase 9, cleaved caspase 9 and MMP-2 were diluted in accordance with the recommended protocols (details are shown in Supplementary Table 3), and the membranes were incubated with these antibodies overnight at 4 °C. Secondary antibodies were added and incubated with the membranes at room temperature for two hours. The membranes were washed with phosphate-buffered saline, and the bands were visualized with a BeyoECL Plus kit (Beyotime) in accordance with the manufacturer’s instructions.

### Pharmacological inhibition of PI3K

To inhibit the activity of PI3K, we treated BGC-823 cells (or *SNHG12*-overexpressing BGC-823 cells) with the PI3K inhibitor LY294002 (Beyotime) dissolved in dimethyl sulfoxide (20 μmol/L). Then, we performed Western blotting, CCK-8, Transwell invasion and cell apoptosis assays by the above methods.

### *In vivo* tumorigenesis assay

Five-week-old male BALB/c nude mice were obtained from SLAC Laboratory Animal Company (Shanghai, China) and fed under specific-pathogen-free conditions. The animal experiments were approved by the Animal Care Committee of Shanghai Sixth People’s Hospital. Si-NC- or si-*SNHG12*-transfected AGS cells (10×10^6^/0.1 mL of serum-free medium) were implanted in the armpit of each mouse. The tumor volume was examined every two days and was calculated by the equation: volume=1/2 (length×width^2^).

### Immunohistochemical staining

A PCNA antibody (Abways, Shanghai, China) was used for immunohistochemical staining of xenograft tumor tissues, as described previously [40]. The positive area of PCNA was calculated with Image-Pro Plus software.

### Statistical analysis

Statistical analyses were conducted with SPSS 21.0 (SPSS, IL, USA) and GraphPad Prism 6 (GraphPad, CA, USA) software. The quantitative variables were analyzed with Student’s *t*-test, while the categorical variables were analyzed with the chi-square test. Univariate and multivariate Cox proportional hazards regression models were used to identify factors associated with the prognosis of GC patients. Overall survival was analyzed with the Kaplan-Meier method and the log-rank test. *P* < 0.05 was considered statistically significant, and all *P* values were two-sided.

## Supplementary Material

Sequences

Supplementary Figures

Supplementary Tables
