# Supplementary material for: The long non-coding RNA SNHG12 promotes gastric cancer by activating the phosphatidylinositol 3-kinase/AKT pathway
Source: Aging (Albany NY). 2019 Dec 5;11(23):10902–22. doi: 10.18632/aging.102493 (PMC6932881; doi:10.18632/aging.102493)
Supplement: Sequences [file aging-11-102493-s001..pdf]

Sequences

catRAPID:
http://service.tartaglialab.com/page/catrapid_group

PRIDB: http://pridb.gdcb.iastate.edu/RPISeq/

The sequence of lncRNA SNHG12:

CCTTTCTCCCCGCCGCATTCCCGGTGTCGACTTA
CTAGCTGCAAGCCTCTGCCTGCCTTCCTGCGCG
CCGTTCCCCGCTAGTCGCTGCTGCTGGCGCGCA
CTCGCCGGGTTTTTCCTCCCACGGCCTCGAGAT
GGTGGTGAATGTGGCACGGAGGAGCCGGGCCT
TCCAACCCGGTGGGCCCGAGCTCCGAAAGGCC
CCCTCGGCAGTGAGAGGGGCGGGAGCCCGCGG
GGGCCGCGCCCTTCTCTCGCTTCGGACTGCGCA
ACGCTGCGCTCTGGGCTGACAGGCGGATAAAA
CGGTCCCATCAAGACTGAGAAAAAGCACACCA
GCTATTGGCACAGCGTGGGCAGTGGGGCCTAC
AGGATGACTGACTTAGTCTACAGAGATCCCGGC
GTACTTAAGCAGATGAAGACTCTTAAGATGAC
AGAAGGTGATTTTTCTGGTGATCGAGGACTTCC
GGGGTAATGACAGTGATGAAATGCAGGGGACC
TGGTTGCCCCCAAGTTTCCTGGCAGTGTGTGAT
ACTGAGGAGGTGAGCTTGTTTCTGGAGCTGTGC
TTTAAGATTCATGTTACATGTAAAGCTGTCCTC
ATTTGTGACTATGGACCTATGGAGTTGGGACAA
TCTCTATGGGAAGCAGAAGGCAAGGACCCCGG
TCATTTTAGGTAGAAACAACAGCATGCTAATGC
AAAAAATTATGCAGTGTGCTACTGAACTTCAGA
GGTGATCAATAAAAGAAGAATAAAAAGACTAA
TAAAAGTAAAAAAAAAAAAAAAAAAAA

The sequence of PI3K (PIK3R1):

MSAEGYQYRALYDYKKEREEDIDLHLGDILTVN
KGSLVALGFSDGQEARPEEIGWLNGYNETTGERG
DFPGTYVEYIGRKKISPPTPKPRPPRPLPVAPGSSK
TEADVEQQALTLPDLAEQFAPPDIAPPLLIKLVEAI
EKKGLECSTLYRTQSSSNLAELRQLLDCDTPSVDL
EMIDVHVLADAFKRYLLDLPNPVIPAAVYSEMISL
APEVQSSEEYIQLLKKLIRSPSIPHQYWLTLQYLLK
HFFKLSQTSSKNLLNARVLSEIFSPMLFRFSAASD
NTENLIKVIEILISTEWNERQPAPALPPKPPKPTTV
ANNGMNNNMSLQDAEWYWGDISREEVNEKLRD
TADGTFLVRDASTKMHGDYTLTLRKGGNNKLIKI
FHRDGKYGFSDPLTFSSVVELINHYRNESLAQYNP
KLDVKLLYPVSKYQQDQVVKEDNIEAVGKKLHE
YNTQFQEKSREYDRLYEEYTRTSQEIQMKRTAIE
AFNETIKIFEEQCQTQERYSKEYIEKFKREGNEKEI
QRIMHNYDKLKSRISEIIDSRRRLEEDLKKQAAEY
REIDKRMNSIKPDLIQLRKTRDQYLMWLTQKGVR
QKKLNEWLGNENTEDQYSLVEDDEDLPHHDEKT
WNVGSSNRNKAENLLRGKRDGTFLVRESSKQGC
YACSVVVDGEVKHCVINKTATGYGFAEPYNLYS
SLKELVLHYQHTSLVQHNDSLNVTLAYPVYAQQ
RR
