# Supplementary material for: The long non-coding RNA SNHG12 promotes gastric cancer by activating the phosphatidylinositol 3-kinase/AKT pathway
Source: Aging (Albany NY). 2019 Dec 5;11(23):10902–22. doi: 10.18632/aging.102493 (PMC6932881; doi:10.18632/aging.102493)
Supplement: Supplementary Figures [file aging-11-102493-s003..pdf]

SUPPLEMENTARY FIGURES

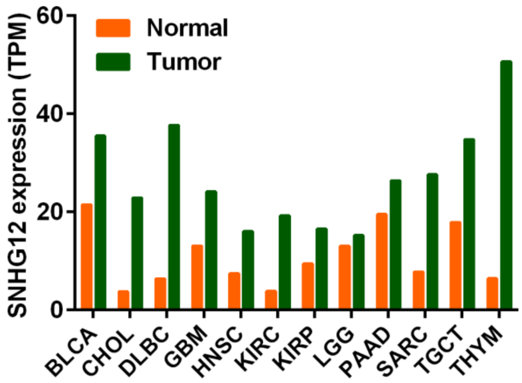

Supplementary Figure 1. SNHG12 was upregulated in various malignancies. The data is the RNA sequencing data downloaded from the GEPIA (Gene Expression Profiling Interactive Analysis, http://gepia.cancer-pku.cn/index.html), and the unit for the expression level of SNHG12 is TPM (Transcripts Per Kilobase of exon model per Million mapped reads).

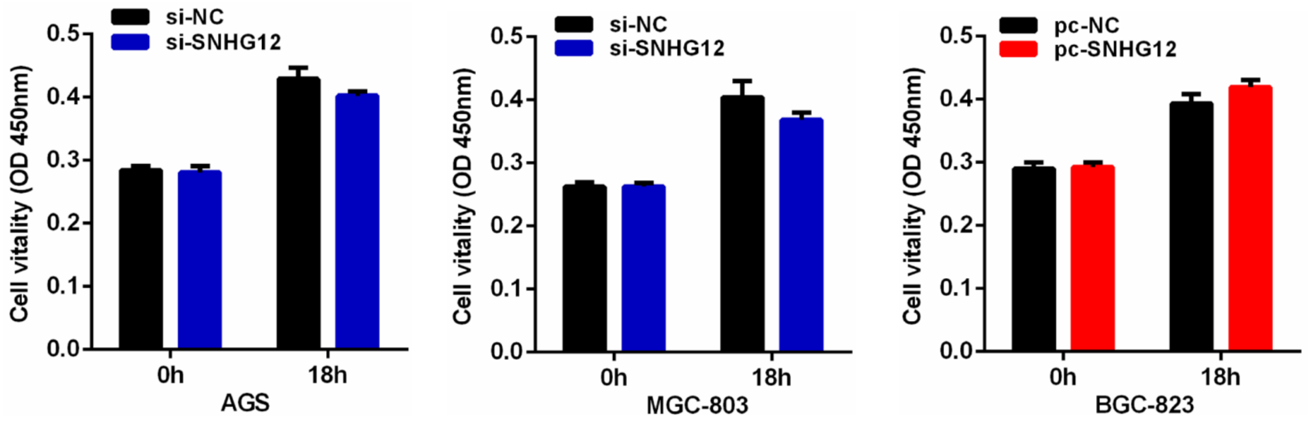

Supplementary Figure 2. Proliferative activities at 18h of AGS and MGC-803 cells transfected with si-SNHG12 and BGC-823 cells transfected with pc-SNHG12 were detected by CCK-8 assay.

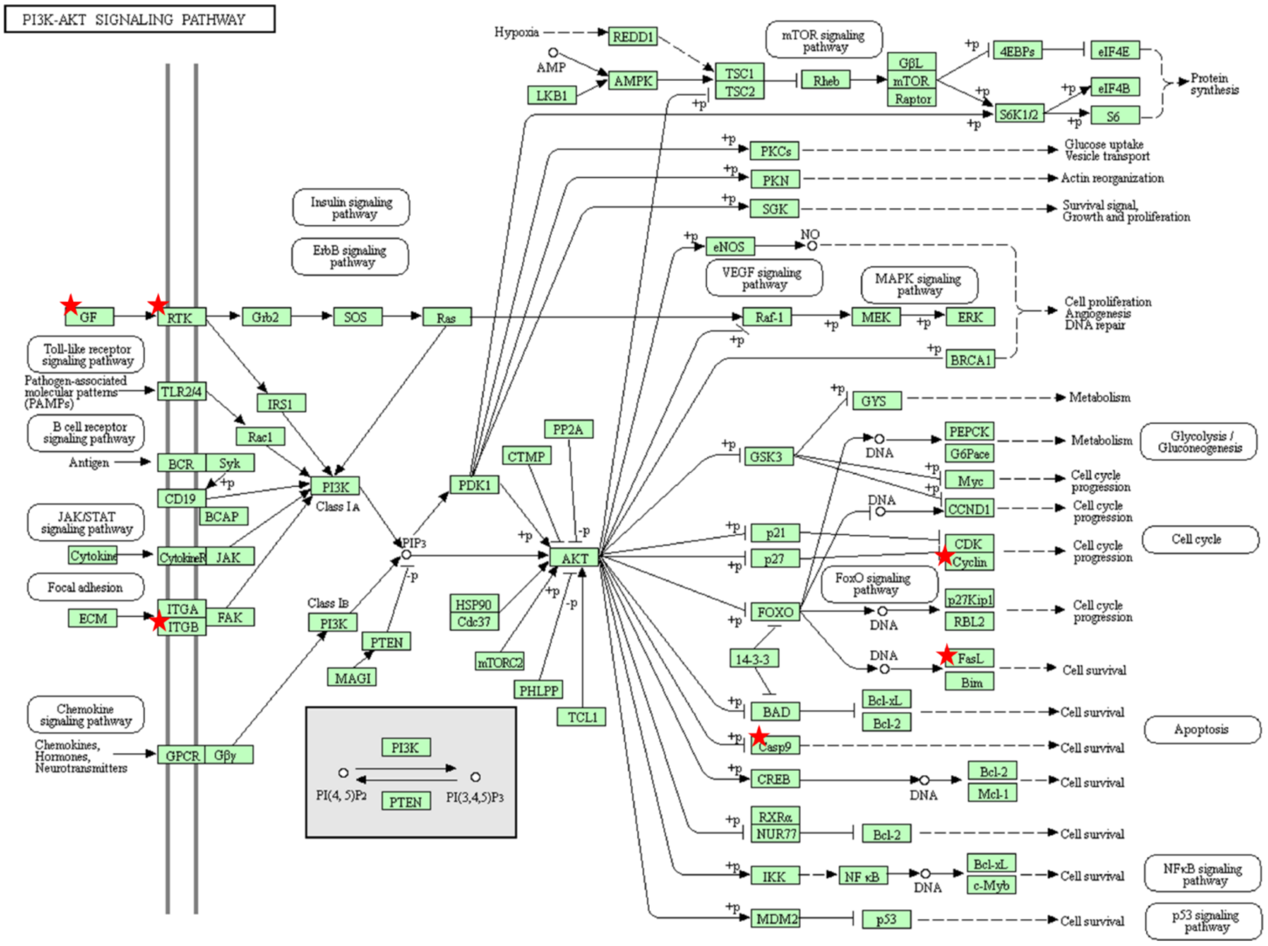

Supplementary Figure 3. The differentially expressed genes enriched in the PI3K/AKT pathway analyzed by DAVID website.
