# Supplementary material for: The long non-coding RNA SNHG12 promotes gastric cancer by activating the phosphatidylinositol 3-kinase/AKT pathway
Source: Aging (Albany NY). 2019 Dec 5;11(23):10902–22. doi: 10.18632/aging.102493 (PMC6932881; doi:10.18632/aging.102493)
Supplement: Supplementary Tables [file aging-11-102493-s002..pdf]

SUPPLEMENTARY TABLES

Supplementary Table 1. The correlation between SNHG12 and clinic-pathological factors of GC patients (Tissue microarray).

Characteristics	Number of cases	Expression of SNHG12		*P* value*
		low	high	
Gender				0.309
male	56	22	34	
female	19	5	14	
Age				0.884
≤60	23	8	15	
>60	52	19	33	
Histological grade				0.307
Low	50	20	30	
middle + high	25	7	18	
Tumor invasion depth				**0.021***
T1	6	5	1	
T2 + T3 + T4	69	22	47	
Lymph node metastasis				0.814
N0	21	8	13	
N1 + N2 + N3	54	19	35	
Distant metastasis				0.594
M0	73	26	47	
M1	2	1	1	
TNM stage				**0.013***
I+II	33	17	16	
III+ IV	42	10	32	

*chi-square test, * *P*< 0.05, statistical significant results (in bold)

Supplementary Table 2. Univariate and multivariate analysis of clinic-pathological factors for OS in GC patients (Tissue microarray).

Risk factors	Univariate analysis			Multivariate analysis		
	HR	*P* value	95% CI	HR	*P* value	95% CI
SNHG12 expression (low, high)	1.957	**0.035***	1.049 ~ 3.652	1.647	**0.025***	0.870~3.118
Age (≤ 60, > 60)	2.273	**0.021***	1.130 ~ 4.572	2.594	**0.010***	1.257~5.354
Lymph node metastasis (N0, N1 + N2 + N3)	2.328	**0.023***	1.124 ~ 4.821			
TNM stage (I+II, III+IV)	3.453	< **0.001****	1.812 ~ 6.578	3.301	**0.003****	1.506~7.237
Tumor invasion depth (T1, T2 + T3 + T4)	3.235	0.105	0.783~ 13.357			
Distant metastasis (M0, M1)	2.370	0.235	0.571 ~ 9.830			
Histological grade (low, middle + high)	0.709	0.272	0.385 ~ 1.308			
Gender (female, male)	1.160	0.666	0.591 ~ 2.278			

HR hazard ratio, * $P < 0.05$, ** $P < 0.01$, statistical significant results (in bold)

Supplementary Table 3. Details of primary antibodies.

Name	Brand	Item number	Dilution	Molecular weight	Source	Clone
PI3K	Bioss	bs-0128R	1:1000	85 kD	Rabbit	Polyclonal
p-PI3K	Bioss	bs-3332R	1:1000	85 kD	Rabbit	Polyclonal
AKT	Affinity	AF6261	1:1000	55 kDa	Rabbit	Polyclonal
p-AKT	Affinity	AF0016	1:1000	60 kDa	Rabbit	Polyclonal
MEK 1/2	Abways	CY5168	1:1500	44 kDa	Rabbit	Monoclonal
p-MEK 1/2	Bioss	bs-3270R	1:1000	43 kD	Rabbit	Polyclonal
ERK 1/2	Abways	CY5487	1:1000	42,44 kDa	Rabbit	Polyclonal
p-ERK 1/2	Abways	CY5277	1:1000	42,44 kDa	Rabbit	Polyclonal
cyclin D1	Abways	CY5404	1:2000	34 kDa	Rabbit	Monoclonal
caspase-9<br>cleaved caspase-9	Abways	CY5782	1:1000	46,39,37,35 kDa	Rabbit	Polyclonal
MMP-2	Abways	CY5189	1:800	72 kDa	Rabbit	Monoclonal
GAPDH	Bioss	bs-10900R	1:1000	38 kDa	Rabbit	Polyclonal
PCNA	Abways	AB0051	1:50	29 kDa	Rabbit	Monoclonal
